# Crystal structure of (1*S*,2*R*)-6,6-dimethyl-4,8-dioxo-2-phenyl­spiro­[2.5]octane-1-carbaldehyde

**DOI:** 10.1107/S205698901600164X

**Published:** 2016-01-30

**Authors:** Saloua Chelli, Konstantin Troshin, Sami Lakhdar, Herbert Mayr, Peter Mayer

**Affiliations:** a Ludwig-Maximilians-Universität, Department, Butenandtstrasse 5–13, 81377 München, Germany; b Department Chemie und Biochemie, Ludwig-Maximilians Universität, Butenandtstrasse 5–13 (Haus F), D-81377 München, Germany

**Keywords:** crystal structure, substituted spiro-cyclo­propanes, weak C—H⋯O and C—H⋯π inter­actions

## Abstract

In the title compound, C_17_H_18_O_3_, the two non-spiro C atoms of the cyclo­propane ring bear a formyl and a phenyl substituent which are *trans*-oriented. In the crystal, mol­ecules are linked by weak C—H⋯O and C—H⋯π contacts resulting in a three-dimensional supra­molecular structure.

## Abstract 2

The title spiro-compound bears *trans*-bound formyl and phenyl substituents at the cyclo­propane ring. In the crystal, mol­ecules are linked by weak C—H⋯O and C—H⋯π contacts, resulting in a three-dimensional supra­molecular structure.

## Chemical context   

Apart from synthetic transformations, cyclo­propane derivatives have attracted inter­est because of their biological and pharmaceutical applications (Wessjohann *et al.*, 2003 ▸). They are present in numerous natural products and have been used extensively as reactive inter­mediates for the formation of complex structures (Reissig & Zimmer, 2003 ▸; Thibodeaux *et al.*, 2012 ▸). During our studies on the reactivities of iodo­nium ylides, we have developed a new method for the synthesis of substituted spiro-cyclo­propanes by the organocatalytic reaction of α,β-unsaturated aldehydes with iodo­nium ylides. The title compound was obtained by the reaction of the cinnamaldehyde-derived iminium ion derived from MacMillan first generation catalyst and the dimedone-derived phenyl­iodo­nium ylide.
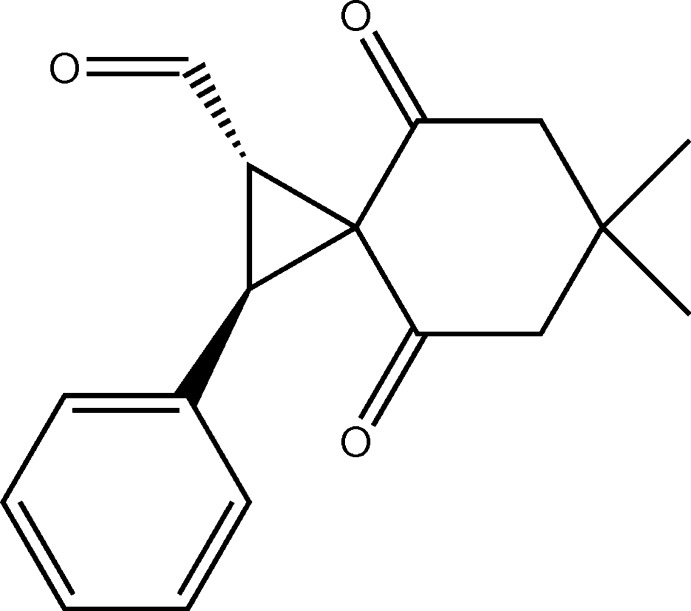


## Structural commentary   

**Figure 1:**
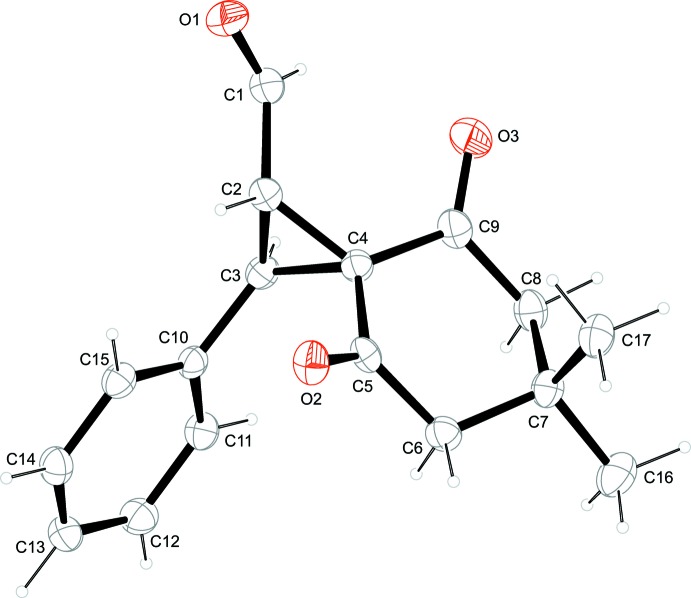
A view of the mol­ecular structure of the title compound, showing the atom labelling. Displacement ellipsoids are drawn at the 50% probability level.

The mol­ecular structure of the title compound is depicted in Fig. 1 ▸. The central cyclo­propane ring shares the spiro atom C4 with a cyclo­hexane ring system while atoms C2 and C3 bear a formyl and a phenyl substituent, respectively. The latter two substituents are *trans*-oriented regarding the plane of the cyclo­propane ring. The angles in the three-membered ring range from 58.80 (13)° (C2—C4—C3) to 61.67 (13)° (C3—C2—C4) being close to the ideal value of 60° for such a ring. The six-membered ring containing the spiro atom C4 and ring atoms C5–C9 adopts a chair conformation with a puckering amplitude *Q* of 0.491 (2) Å and *θ* = 16.8 (2)°, which indicates a slight deviation from an ideal chair conformation with *θ* = 0°. The plane of the central cyclo­propane ring forms dihedral angles of 66.89 (16) and 89.33 (16)°, respectively, with the plane of the phenyl ring and the mean plane of the cyclo­hexane ring [maximum deviation from this plane is 0.272 (2) Å for atom C7]. The latter two planes form a dihedral angle of 64.15 (10)°. The plane of the formyl group, consisting of atoms C1, H1 and O1, is almost normal to the cyclo­propane ring with a dihedral angle of 81.3 (3)°.

## Supra­molecular features   

**Figure 2:**
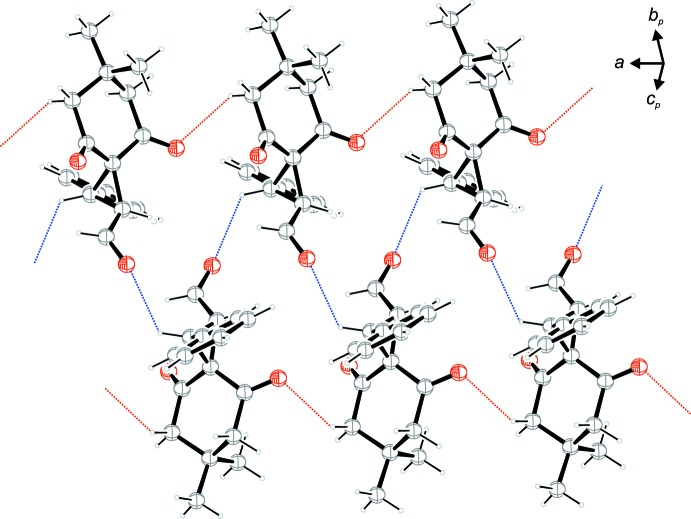
A view of the double strands along [100] formed by two different weak C—H⋯O contacts (red and blue dashed lines; see Table 1 ▸ for details).

**Figure 3:**
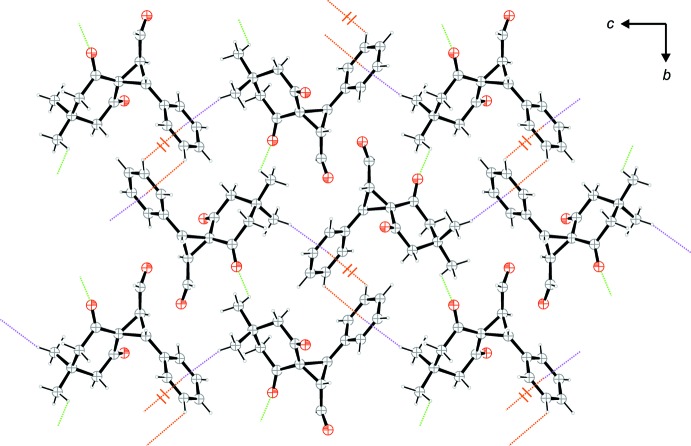
The packing established by weak C—H⋯O contacts (green dotted lines) C—H⋯π contacts (violet and orange dotted lines) viewed along [100]; see Table 1 ▸ for details. Slashed dotted lines indicate bonds to a symmetry-related mol­ecule.

The crystal packing of the title compound shows weak C—H⋯O and C—H⋯π inter­actions (Table 1 ▸ and Figs. 2 ▸ and 3 ▸). Two of the three different C—H⋯O contacts lead to the formation of double strands along [100]; Fig. 2 ▸. Single strands are formed by C8—H8*B*⋯O2 contacts (red dotted lines) which are further linked to double strands along the 2_1_-screw axes along [100] by C3—H3⋯O1 contacts (blue dotted lines). The remaining C—H⋯O as well as the C—H⋯π inter­actions are displayed in Fig. 3 ▸, which shows details of the crystal packing viewed along [100]. Strands along [010] are established by C16—H16*B*⋯O3 contacts (green dotted lines). These strands are linked by two different C—H⋯π contacts (Table 1 ▸), both of which have one of the two sides of the phenyl ring (C10–C15) as π-acceptor (*Cg* is the centroid of this ring). Along [100] the strands are linked by C12—H12⋯*Cg^i^* ^v^ contacts (orange dotted lines) while along [001] the links are established by C17—H17*B*⋯*Cg* ^v^ inter­actions (violet dotted lines) enclosing angles between the C—H bond and the plane of the π-system of *ca* 39° and 75° respectively. As a result of these inter­actions, a three-dimensional supra­molecular structure is formed.

## Database survey   

Structures of spiro­[2.5]octane and 4-oxo-spiro­[2.5]octane derivatives are numerous; however, there are merely two different structures featuring the 6,6-dimethyl-4,8-dioxo-spiro­[2.5]octane moiety as is found in the title compound, namely 6,6-dimethyl-4,8-dioxo-1,1,2,2-tetra­cyano-spiro­(2,5)octane 1,4-dioxane solvate (NOSMIR; Kayukova *et al.*, 1998 ▸) and *trans*-1,2-bis­(meth­oxy­carbon­yl)-6,6-di­methyl­spiro­(2.5)octane-4,8-dione (GUHCUI; Maghsoodlou *et al.*, 2009 ▸). Two more structures feature the 4,8-dioxo-spiro­[2.5]octane building unit, namely. tris­piro(2.1.2.1.2.1)dodecane-4,8,12-trione (DAZVEF; Hoffmann *et al.*,1985 ▸) and (2R*)-1,1-dichloro-6,6-dimethyl-2-[(1′S*)-1′-nitro­eth­yl]spiro­[2.5]octane-4,8-dione (YILXIC; Barkov *et al.*, 2013 ▸). In NOSMIR, each of the two non-spiro-cyclo­propane C atoms bears two cyano groups while in GUHCUI each of the C atoms bears a hydrogen atom and a meth­oxy­lcarbonyl group. The latter substituents are, as in the title compound, *trans*-oriented with respect to the plane of the cyclo­propane ring.

## Synthesis and crystallization   

A 10 ml round-bottomed flask equipped with a magnetic stirring bar was charged with a solution of the (*S*,*E*)-5-benzyl-2,2,3-trimethyl-4-oxo-1-[(*E*)-3-phenyl­allyl­idene]-imidazolidin-1-ium hexa­fluoro­phosphate (239 mg, 0.5 mmol, 1eq) and phenyl­iodo­nium-4,4-di­methyl­cyclo­hexane-2,6-dione (171 mg, 0.5 mmol, 1eq) in aceto­nitrile (5 ml). After 24 h stirring at ambient temperature, water (10 ml) was added. The aqueous phase was extracted with CH_2_Cl_2_ (15 ml). The organic layers were combined, washed with brine, and dried over MgSO_4_. After evaporation of the solvent under vacuum, the crude product was purified by column chromatography (*n*-penta­ne/Et2O: 7/3 and 6/4) to give the title compound (98 mg, 0.362 mmol, 72%) as colourless crystals (m.p. 397–399 K).

## Refinement   

Crystal data, data collection and structure refinement details are summarized in Table 2 ▸. C-bound H atoms were positioned geometrically (C—H = 0.95–1.00 Å) and treated as riding on their parent atoms with *U*_iso_(H) = 1.5*U*_eq_(C-meth­yl) and 1.2*U*_eq_(C) for other H atoms. The methyl groups were allowed to rotate along the C—C bonds to best fit the experimental electron density. As a result of the absence of anomalous scatterers and high angle data, the Flack test results can be considered meaningless. The synthesis resulted in a racemic mixture, hence the structure was refined as an inversion twin.

## Supplementary Material

Crystal structure: contains datablock(s) I, global. DOI: 10.1107/S205698901600164X/su5275sup1.cif

Structure factors: contains datablock(s) I. DOI: 10.1107/S205698901600164X/su5275Isup2.hkl

Supporting information file. DOI: 10.1107/S205698901600164X/su5275Isup3.cml

CCDC reference: 1450224

Additional supporting information:  crystallographic information; 3D view; checkCIF report

## Figures and Tables

**Table 1:** Hydrogen-bond geometry (Å, °) *Cg* is the centroid of the C10–C15 phenyl ring.

*D*—H⋯*A*	*D*—H	H⋯*A*	*D*⋯*A*	*D*—H⋯*A*
C3—H3⋯O1^i^	1.00	2.50	3.159 (3)	123
C8—H8*B*⋯O2^ii^	0.99	2.58	3.365 (2)	137
C16—H16*C*⋯O3^iii^	0.98	2.55	3.271 (3)	131
C12—H12⋯*Cg* ^iv^	0.95	2.97	3.688 (2)	133
C17—H17*B*⋯*Cg* ^v^	0.98	2.97	3.916 (2)	163

Symmetry codes: (i) 
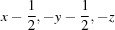
; (ii) 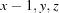; (iii) 
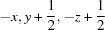
; (iv) 
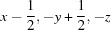
; (v) 
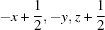
.

**Table 2:** Experimental details

Crystal data	
Chemical formula	C_17_H_18_O_3_
*M*_r_	270.31
Crystal system, space group	Orthorhombic, *P*2_1_2_1_2_1_
Temperature (K)	173
*a*, *b*, *c* (Å)	5.8831 (1), 12.9095 (4), 18.5655 (5)
*V* (Å^3^)	1410.01 (6)
*Z*	4
Radiation type	Mo *K*α
μ (mm^−1^)	0.09
Crystal size (mm)	0.24 × 0.08 × 0.04

Data collection	
Diffractometer	Nonius KappaCCD
No. of measured, independent and observed [*I* > 2σ(*I*)] reflections	11599, 3227, 2714
*R*_int_	0.042
(sin θ/λ)_max_ (Å^−1^)	0.650

Refinement	
*R*[*F* ^2^ > 2σ(*F* ^2^)], *wR*(*F* ^2^), *S*	0.037, 0.085, 1.05
No. of reflections	3227
No. of parameters	183
H-atom treatment	H-atom parameters constrained
Δρ_max_, Δρ_min_ (e Å^−3^)	0.15, −0.17
Absolute structure	Refined as a perfect inversion twin
Absolute structure parameter	0.5

Computer programs: *COLLECT* (Hooft, 2004 ▸), *DENZO* and *SCALEPACK* (Otwinowski & Minor, 1997 ▸), *SIR97* (Altomare *et al.*, 1999 ▸), *SHELXL2014* (Sheldrick, 2015 ▸), *ORTEPIII* (Burnett & Johnson, 1996 ▸) and *PLATON* (Spek, 2009 ▸).

## supplementary crystallographic information

### Crystal data

**Table:**

C_17_H_18_O_3_	*D*_x_ = 1.273 Mg m^−^^3^
*M**_r_* = 270.31	Mo *K*α radiation, λ = 0.71073 Å
Orthorhombic, *P*2_1_2_1_2_1_	Cell parameters from 5895 reflections
*a* = 5.8831 (1) Å	θ = 3.1–27.5°
*b* = 12.9095 (4) Å	µ = 0.09 mm^−^^1^
*c* = 18.5655 (5) Å	*T* = 173 K
*V* = 1410.01 (6) Å^3^	Rod, colourless
*Z* = 4	0.24 × 0.08 × 0.04 mm
*F*(000) = 576	

### Data collection

**Table:**

Nonius KappaCCD diffractometer	2714 reflections with *I* > 2σ(*I*)
Radiation source: FR591 rotating anode generator	*R*_int_ = 0.042
Detector resolution: 9 pixels mm^-1^	θ_max_ = 27.5°, θ_min_ = 3.2°
CCD; rotation images; thick slices scans	*h* = −7→7
11599 measured reflections	*k* = −16→16
3227 independent reflections	*l* = −24→23

### Refinement

**Table:**

Refinement on *F*^2^	Hydrogen site location: inferred from neighbouring sites
Least-squares matrix: full	H-atom parameters constrained
*R*[*F*^2^ > 2σ(*F*^2^)] = 0.037	*w* = 1/[σ^2^(*F*_o_^2^) + (0.038*P*)^2^ + 0.2287*P*] where *P* = (*F*_o_^2^ + 2*F*_c_^2^)/3
*wR*(*F*^2^) = 0.085	(Δ/σ)_max_ < 0.001
*S* = 1.05	Δρ_max_ = 0.15 e Å^−^^3^
3227 reflections	Δρ_min_ = −0.17 e Å^−^^3^
183 parameters	Absolute structure: Refined as a perfect inversion twin
0 restraints	Absolute structure parameter: 0.5

### Special details

**Table:**

Geometry. All e.s.d.'s (except the e.s.d. in the dihedral angle between two l.s. planes) are estimated using the full covariance matrix. The cell e.s.d.'s are taken into account individually in the estimation of e.s.d.'s in distances, angles and torsion angles; correlations between e.s.d.'s in cell parameters are only used when they are defined by crystal symmetry. An approximate (isotropic) treatment of cell e.s.d.'s is used for estimating e.s.d.'s involving l.s. planes.

### Fractional atomic coordinates and isotropic or equivalent isotropic displacement parameters (Å^2^)

**Table:**

	*x*	*y*	*z*	*U*_iso_*/*U*_eq_	
O1	0.4197 (3)	−0.32107 (11)	0.04731 (9)	0.0342 (4)	
O2	0.7409 (2)	0.01619 (12)	0.11016 (8)	0.0263 (3)	
O3	0.1647 (3)	−0.17348 (13)	0.20019 (9)	0.0393 (4)	
C1	0.3171 (4)	−0.24439 (16)	0.06610 (11)	0.0266 (5)	
H1	0.1638	−0.2515	0.0814	0.032*	
C2	0.4217 (4)	−0.13971 (16)	0.06618 (11)	0.0219 (4)	
H2	0.5742	−0.1348	0.0426	0.026*	
C3	0.2601 (3)	−0.05158 (15)	0.05003 (11)	0.0200 (4)	
H3	0.0965	−0.0725	0.0501	0.024*	
C4	0.3761 (3)	−0.06024 (15)	0.12514 (10)	0.0193 (4)	
C5	0.5547 (3)	0.02063 (16)	0.13825 (10)	0.0201 (4)	
C6	0.4809 (4)	0.10926 (16)	0.18537 (11)	0.0229 (5)	
H6A	0.3774	0.1545	0.1575	0.027*	
H6B	0.6162	0.1508	0.1985	0.027*	
C7	0.3597 (3)	0.07468 (16)	0.25467 (11)	0.0221 (4)	
C8	0.1576 (3)	0.00558 (16)	0.23406 (11)	0.0243 (5)	
H8A	0.0845	−0.0204	0.2786	0.029*	
H8B	0.0443	0.0478	0.2077	0.029*	
C9	0.2249 (4)	−0.08522 (17)	0.18801 (11)	0.0236 (5)	
C10	0.3127 (3)	0.03408 (15)	−0.00113 (10)	0.0186 (4)	
C11	0.1524 (3)	0.11331 (15)	−0.00784 (11)	0.0223 (4)	
H11	0.0168	0.1111	0.0199	0.027*	
C12	0.1900 (4)	0.19527 (16)	−0.05476 (12)	0.0256 (5)	
H12	0.0811	0.2493	−0.0586	0.031*	
C13	0.3866 (4)	0.19845 (16)	−0.09613 (12)	0.0248 (5)	
H13	0.4114	0.2541	−0.1287	0.030*	
C14	0.5461 (4)	0.12027 (16)	−0.08971 (11)	0.0241 (5)	
H14	0.6812	0.1227	−0.1177	0.029*	
C15	0.5103 (3)	0.03793 (15)	−0.04251 (11)	0.0219 (4)	
H15	0.6205	−0.0156	−0.0385	0.026*	
C16	0.2732 (4)	0.17058 (18)	0.29458 (13)	0.0330 (5)	
H16A	0.4007	0.2175	0.3041	0.049*	
H16B	0.2039	0.1495	0.3403	0.049*	
H16C	0.1598	0.2061	0.2649	0.049*	
C17	0.5246 (4)	0.01436 (18)	0.30256 (12)	0.0283 (5)	
H17A	0.5775	−0.0474	0.2769	0.042*	
H17B	0.4470	−0.0065	0.3470	0.042*	
H17C	0.6550	0.0583	0.3145	0.042*	

### Atomic displacement parameters (Å^2^)

**Table:**

	*U*^11^	*U*^22^	*U*^33^	*U*^12^	*U*^13^	*U*^23^
O1	0.0489 (10)	0.0214 (8)	0.0324 (9)	0.0031 (7)	−0.0002 (8)	−0.0022 (7)
O2	0.0174 (7)	0.0365 (9)	0.0249 (8)	−0.0033 (6)	0.0021 (6)	−0.0018 (7)
O3	0.0496 (10)	0.0335 (9)	0.0349 (9)	−0.0186 (8)	0.0107 (8)	0.0005 (7)
C1	0.0348 (12)	0.0242 (11)	0.0208 (11)	−0.0024 (9)	−0.0013 (9)	0.0010 (8)
C2	0.0242 (10)	0.0217 (10)	0.0199 (10)	0.0005 (8)	0.0005 (8)	0.0011 (8)
C3	0.0190 (10)	0.0206 (10)	0.0204 (10)	−0.0011 (8)	−0.0013 (8)	−0.0026 (8)
C4	0.0195 (10)	0.0193 (10)	0.0192 (10)	−0.0014 (8)	−0.0004 (8)	0.0001 (8)
C5	0.0199 (10)	0.0243 (10)	0.0160 (9)	−0.0008 (8)	−0.0016 (8)	0.0049 (8)
C6	0.0222 (10)	0.0229 (10)	0.0236 (11)	−0.0030 (8)	−0.0003 (9)	0.0008 (8)
C7	0.0204 (10)	0.0259 (11)	0.0200 (10)	0.0022 (8)	0.0008 (8)	−0.0018 (8)
C8	0.0197 (10)	0.0320 (11)	0.0213 (11)	−0.0009 (9)	0.0026 (8)	0.0009 (9)
C9	0.0193 (10)	0.0301 (11)	0.0214 (10)	−0.0062 (9)	−0.0006 (8)	0.0014 (8)
C10	0.0202 (10)	0.0197 (9)	0.0160 (9)	−0.0008 (8)	−0.0019 (8)	−0.0023 (8)
C11	0.0203 (10)	0.0253 (10)	0.0214 (11)	0.0017 (8)	0.0001 (8)	−0.0012 (8)
C12	0.0277 (11)	0.0232 (10)	0.0259 (11)	0.0037 (9)	−0.0028 (9)	0.0007 (9)
C13	0.0303 (12)	0.0235 (11)	0.0207 (11)	−0.0019 (9)	−0.0016 (9)	0.0019 (8)
C14	0.0228 (11)	0.0280 (11)	0.0215 (11)	−0.0013 (9)	0.0033 (9)	−0.0004 (9)
C15	0.0213 (10)	0.0223 (10)	0.0220 (11)	0.0028 (8)	−0.0014 (8)	−0.0026 (8)
C16	0.0344 (12)	0.0332 (12)	0.0314 (12)	0.0079 (10)	0.0025 (11)	−0.0077 (10)
C17	0.0290 (11)	0.0332 (12)	0.0226 (11)	0.0050 (10)	−0.0017 (9)	−0.0020 (9)

### Geometric parameters (Å, º)

**Table:**

O1—C1	1.211 (3)	C8—C9	1.504 (3)
O2—C5	1.214 (2)	C8—H8A	0.9900
O3—C9	1.214 (3)	C8—H8B	0.9900
C1—C2	1.485 (3)	C10—C15	1.394 (3)
C1—H1	0.9500	C10—C11	1.396 (3)
C2—C3	1.513 (3)	C11—C12	1.388 (3)
C2—C4	1.524 (3)	C11—H11	0.9500
C2—H2	1.0000	C12—C13	1.389 (3)
C3—C10	1.490 (3)	C12—H12	0.9500
C3—C4	1.557 (3)	C13—C14	1.383 (3)
C3—H3	1.0000	C13—H13	0.9500
C4—C5	1.501 (3)	C14—C15	1.394 (3)
C4—C9	1.503 (3)	C14—H14	0.9500
C5—C6	1.504 (3)	C15—H15	0.9500
C6—C7	1.537 (3)	C16—H16A	0.9800
C6—H6A	0.9900	C16—H16B	0.9800
C6—H6B	0.9900	C16—H16C	0.9800
C7—C17	1.529 (3)	C17—H17A	0.9800
C7—C16	1.530 (3)	C17—H17B	0.9800
C7—C8	1.535 (3)	C17—H17C	0.9800
			
O1—C1—C2	122.5 (2)	C7—C8—H8A	109.0
O1—C1—H1	118.7	C9—C8—H8B	109.0
C2—C1—H1	118.7	C7—C8—H8B	109.0
C1—C2—C3	115.08 (18)	H8A—C8—H8B	107.8
C1—C2—C4	122.71 (18)	O3—C9—C4	121.2 (2)
C3—C2—C4	61.67 (13)	O3—C9—C8	123.29 (19)
C1—C2—H2	115.4	C4—C9—C8	115.47 (18)
C3—C2—H2	115.4	C15—C10—C11	119.18 (18)
C4—C2—H2	115.4	C15—C10—C3	123.40 (17)
C10—C3—C2	123.65 (17)	C11—C10—C3	117.42 (17)
C10—C3—C4	122.23 (16)	C12—C11—C10	120.45 (19)
C2—C3—C4	59.53 (12)	C12—C11—H11	119.8
C10—C3—H3	113.7	C10—C11—H11	119.8
C2—C3—H3	113.7	C11—C12—C13	120.14 (19)
C4—C3—H3	113.7	C11—C12—H12	119.9
C5—C4—C9	115.97 (17)	C13—C12—H12	119.9
C5—C4—C2	117.47 (17)	C14—C13—C12	119.72 (19)
C9—C4—C2	121.17 (17)	C14—C13—H13	120.1
C5—C4—C3	113.71 (16)	C12—C13—H13	120.1
C9—C4—C3	116.85 (16)	C13—C14—C15	120.53 (19)
C2—C4—C3	58.80 (13)	C13—C14—H14	119.7
O2—C5—C4	121.93 (19)	C15—C14—H14	119.7
O2—C5—C6	123.10 (19)	C14—C15—C10	119.97 (19)
C4—C5—C6	114.90 (16)	C14—C15—H15	120.0
C5—C6—C7	113.56 (16)	C10—C15—H15	120.0
C5—C6—H6A	108.9	C7—C16—H16A	109.5
C7—C6—H6A	108.9	C7—C16—H16B	109.5
C5—C6—H6B	108.9	H16A—C16—H16B	109.5
C7—C6—H6B	108.9	C7—C16—H16C	109.5
H6A—C6—H6B	107.7	H16A—C16—H16C	109.5
C17—C7—C16	109.97 (18)	H16B—C16—H16C	109.5
C17—C7—C8	109.90 (17)	C7—C17—H17A	109.5
C16—C7—C8	109.49 (17)	C7—C17—H17B	109.5
C17—C7—C6	109.89 (17)	H17A—C17—H17B	109.5
C16—C7—C6	108.94 (17)	C7—C17—H17C	109.5
C8—C7—C6	108.63 (16)	H17A—C17—H17C	109.5
C9—C8—C7	113.02 (16)	H17B—C17—H17C	109.5
C9—C8—H8A	109.0		
			
O1—C1—C2—C3	147.4 (2)	C5—C6—C7—C8	−55.6 (2)
O1—C1—C2—C4	−141.4 (2)	C17—C7—C8—C9	−64.9 (2)
C1—C2—C3—C10	−134.23 (19)	C16—C7—C8—C9	174.16 (17)
C4—C2—C3—C10	110.6 (2)	C6—C7—C8—C9	55.3 (2)
C1—C2—C3—C4	115.2 (2)	C5—C4—C9—O3	−141.2 (2)
C1—C2—C4—C5	154.52 (19)	C2—C4—C9—O3	12.2 (3)
C3—C2—C4—C5	−102.42 (19)	C3—C4—C9—O3	80.3 (3)
C1—C2—C4—C9	1.5 (3)	C5—C4—C9—C8	38.1 (2)
C3—C2—C4—C9	104.5 (2)	C2—C4—C9—C8	−168.43 (18)
C1—C2—C4—C3	−103.1 (2)	C3—C4—C9—C8	−100.3 (2)
C10—C3—C4—C5	−4.1 (2)	C7—C8—C9—O3	131.5 (2)
C2—C3—C4—C5	108.85 (19)	C7—C8—C9—C4	−47.8 (2)
C10—C3—C4—C9	135.3 (2)	C2—C3—C10—C15	4.1 (3)
C2—C3—C4—C9	−111.8 (2)	C4—C3—C10—C15	76.7 (2)
C10—C3—C4—C2	−112.9 (2)	C2—C3—C10—C11	−176.62 (18)
C9—C4—C5—O2	145.1 (2)	C4—C3—C10—C11	−104.1 (2)
C2—C4—C5—O2	−9.3 (3)	C15—C10—C11—C12	−0.4 (3)
C3—C4—C5—O2	−75.2 (2)	C3—C10—C11—C12	−179.71 (18)
C9—C4—C5—C6	−37.9 (2)	C10—C11—C12—C13	0.8 (3)
C2—C4—C5—C6	167.68 (17)	C11—C12—C13—C14	−0.8 (3)
C3—C4—C5—C6	101.85 (19)	C12—C13—C14—C15	0.5 (3)
O2—C5—C6—C7	−135.3 (2)	C13—C14—C15—C10	−0.2 (3)
C4—C5—C6—C7	47.7 (2)	C11—C10—C15—C14	0.1 (3)
C5—C6—C7—C17	64.7 (2)	C3—C10—C15—C14	179.37 (18)
C5—C6—C7—C16	−174.76 (18)		

### Hydrogen-bond geometry (Å, º)

Cg is the centroid of the C10–C15 phenyl ring.

**Table:**

*D*—H···*A*	*D*—H	H···*A*	*D*···*A*	*D*—H···*A*
C3—H3···O1^i^	1.00	2.50	3.159 (3)	123
C8—H8*B*···O2^ii^	0.99	2.58	3.365 (2)	137
C16—H16*C*···O3^iii^	0.98	2.55	3.271 (3)	131
C12—H12···*Cg*^iv^	0.95	2.97	3.688 (2)	133
C17—H17*B*···*Cg*^v^	0.98	2.97	3.916 (2)	163

Symmetry codes: (i) *x*−1/2, −*y*−1/2, −*z*; (ii) *x*−1, *y*, *z*; (iii) −*x*, *y*+1/2, −*z*+1/2; (iv) *x*−1/2, −*y*+1/2, −*z*; (v) −*x*+1/2, −*y*, *z*+1/2.
